# An Overview of Seafood Supply, Food Safety and Regulation in New South Wales, Australia

**DOI:** 10.3390/foods6070052

**Published:** 2017-07-19

**Authors:** Malik A. Hussain, Themy Saputra, Elizabeth A. Szabo, Bruce Nelan

**Affiliations:** NSW Food Authority, Department of Primary Industries, Newington, NSW 2127, Australia; Themy.Saputra@foodauthority.nsw.gov.au (T.S.); Lisa.Szabo@foodauthority.nsw.gov.au (E.A.S.); Bruce.Nelan@foodauthority.nsw.gov.au (B.N.)

**Keywords:** seafood, sources, wild catch, food safety, foodborne outbreaks, NSW food authority

## Abstract

Seafood consumption is increasing in Australia, especially in New South Wales (NSW). Average per capita seafood consumption in NSW is higher than the national average. Seafood supply in NSW comes from domestic (wild catch and aquaculture) and overseas (seafood imports) sources. The contribution of wild catch and aquaculture in domestic seafood production (2012–2013) was 73.42% and 26.52%, respectively. Seafood-associated foodborne illness outbreaks are not common and on an average four outbreaks occur each year in NSW. Most of the outbreaks in 2015 and 2016 were related to ciguatera poisoning. The regulation of the seafood industry and the management of food safety is an example of the coordinated work of multiple government agencies and organizations in which NSW Food Authority is responsible for managing the overall risks through the Seafood Safety Scheme. Overall, seafood supply in NSW is of high quality and poses low food safety risk to consumers.

## 1. Introduction

Seafood is one the favourite foods of Australians. Its consumption rate is increasing each year. Current per capita seafood consumption is over 15 kg, which has increased from 13 kg in 2000. Fisheries are Australia’s fifth most valuable food industry after meat, grains and oilseeds, fruit and vegetables, and milk. The Australian seafood industry has two major components: wild capture and aquaculture. In terms of world seafood production, Australia ranks 46th with a total production of 241,123 tons in 2009–2010 [1].

The total estimated Australian consumption of seafood products was around 345,000 tonnes in 2012–2013 [2]. Only one-third of seafood demand is met from the domestic sources. Imported seafood products accounted for 66% of domestic consumption. Australia imports lower cost seafood products such as frozen fillets, frozen prawns and canned fish from Thailand, New Zealand and China. Australia exports high value products such as rock lobster, abalone and tuna to Japan, Hong Kong and the USA.

According to the Australian Health Survey (2011–2012), the average consumption of seafood in New South Wales (NSW) was 17.5 kg (16.6 kg Australian national average) and 19.2 kg (17.8 kg Australian national average) per capita per year by male and female consumers, respectively [3]. NSW fishing and aquaculture industries offer consumers diverse and high quality seafood. Finfish, prawns, lobster, oysters and crabs are the popular species in NSW. Wild catch and aquaculture are also the major fish supply chains of the domestic products in NSW. Recreational catch is not permitted to be sold; however, it must be noted that recreational fishing makes a significant contribution to NSW economy in terms of tourism.

A range of food safety hazards, scombroid poisoning, ciguatoxin, algal biotoxins, microbiological contamination, and environmental pollutants can be present in different seafood products sold in NSW. The management of food safety hazards associated with seafood, such as minimising the risk of scombroid poisoning, requires general food safety control measures and the use of appropriate storage temperatures. The Seafood Safety Scheme of the NSW Food Authority (the Food Authority) requires businesses processing seafood to implement a food safety program to ensure appropriate process control measures for these hazards.

This article provides a brief overview of seafood sources, food safety concerns, foodborne illness outbreaks and regulation in NSW, Australia.

## 2. Seafood Sources in NSW and Food Safety Hazards

More than two-thirds of domestic seafood production is from marine sources (wild catch) in NSW. Table 1 summarizes key features of the NSW seafood industry. The NSW commercial fishing industry is a dynamic network of skilled businesses which are carefully managed. The wild catch commercial fishing managed under the NSW jurisdiction is worth more than Aus$90 million at first point of sale [4]. The NSW commercial fishing industry is actively working to address the challenges of sustainable production and to improve environmental performance [4].

Freshwater ecosystems are very vulnerable to environmental contamination and invasion by aquatic pests and weeds. NSW inland waterways are known to experience blue green algal blooms. Non-native fish have been accidentally or deliberately introduced into NSW waterways since European settlement and some native Australian fish have been taken out of their natural habitats for recreational fishing enhancement or aquaculture [6]. Freshwater finfish species such as trout and Murray cod (*Maccullochella peelii*) are found in rivers and in freshwater lakes, ponds and dams in NSW. In general, Australian freshwater reservoirs are clean and pose minimum risk as a source of hazards in seafood harvested. In commercial terms, freshwater fish represent a very minor segment (less than 0.5% of total commercial wild catch) of total fisheries products in NSW. Most freshwater fishing is recreational.

Aquaculture is a growing source of fish and seafood and has a different profile of hazards (i.e., environmental pollutants, biotoxins and norovirus) that can contaminate products [7]. Currently, its contribution is more than a quarter of domestic seafood supply and this share is increasing each year. For example, aquaculture production was 26.52% in 2013 and 29% in 2015 of the total seafood production in NSW [5].

In general, important food safety hazards associated with seafood supply in NSW include: scombroid poisoning, ciguatoxin and norovirus associated with wild catch; microbiological contamination and histamine detection in imported seafood; and potential environmental pollutants, algal biotoxins and norovirus in aquaculture products.

## 3. Seafood Associated Outbreaks in NSW

Generally, NSW seafood supply is of good quality and poses minimum health risk to the consumers. On average, about four outbreaks of illness per annum were associated with seafood (domestic and imported) consumption in NSW from 2005 to 2015, whereas the average number of reported egg related foodborne illness outbreaks was more than 8 per year in NSW from 2009 to 2014 [8]. A review of foodborne illness in the USA from 2004 to 2013 published by Center for Science in the Public Interest [9] showed that seafood was responsible for the second-most number of outbreaks (541) with about 10 illnesses per outbreak.

NSW foodborne illness outbreak data for 2005–2015 (Table 2) showed 308 cases (about seven illnesses per outbreak) and 45 hospitalisations linked to consumption of seafood. Further breakdown of the outbreak data revealed 65.11%, 23.25% and 11.63% outbreaks were linked to finfish, shellfish and crustaceans, respectively. The data also showed that one-quarter (25.58%) of the total outbreaks were due to scombroid poisoning. Knope et al. [10] reported 57 histamine (scombroid) fish poisoning outbreaks in Australia from 2001 to 2013 caused by scombridae fish (Tuna, 57%) and non-scombridae fish (mahi-mahi, 14%; yellowtail kingfish/kingfish 7%). They reported that the majority of the histamine (scombroid) fish poisoning outbreaks (36.84%, 21 out of 57) occurred in NSW.

A brief description of recent outbreaks (2 is given below):

(1) In February 2015, seven cases of scombroid poisoning were identified. All victims had onset of symptoms (red face, headache, tingling, sweating, vomiting and palpitations) within 10–15 min after consuming tuna salads from the same local food outlet. Investigation conducted by the Food Authority implicated imported canned tuna and thus, a trade recall was carried out [11]. According to OzFoodNet (a health network to enhance the surveillance of foodborne diseases in Australia), there were 2–3 outbreaks each year involving foods imported into Australia [12].
(2) In April 2015, four persons were affected by ciguatera poisoning after eating locally caught Spanish mackerel at a private function and one was hospitalised [11].
(3) In June 2015, an outbreak of *Salmonella* Agona from tuna sushi rolls affected three people with no hospitalization recorded. Investigation of the incident revealed that samples from one of the sushi outlets were also positive for *S*. Agona. Further investigation and analysis suggested that the source of the *S.* Agona was chicken meat and the outbreak was caused by cross contamination between the chicken and the tuna sushi prepared by the businesses [11].
(4) In September 2015, three people were reported to have ciguatera poisoning after consumption of a red throat emperor fish. The victims were members of a single family who ate the fish that was purchased whole from a local fish market, cleaned and eviscerated in-store, cooked and consumed on the same day. The fish was caught off a regularly fished seamount off the Queensland coast [13].
(5) In 2016, two incidents of ciguatera poisoning were reported. The first incident affected three people after consuming a Spanish mackerel caught off the coast of Crowdy Head in March and the second incident affected one person who consumed a Spanish mackerel caught off the coast at Crescent Head in April. In both cases, the fish consumed were caught by recreational fishermen. In response to these incidents, the NSW Food Authority advised fishers to avoid eating Spanish mackerel above 10 kg, as advised by NSW industry experts. Consumption of large Spanish mackerel poses an increased risk of ciguatera poisoning [14].

Most recent food poisoning outbreaks (2015 and 2016) associated with consumption of seafood that occurred in NSW were related to ciguatera poisoning. However, a relatively high incidence of ciguatera poisoning has been reported in Queensland [15]. Only a small volume of reef fish from Queensland and other problem areas is sold in NSW. There have been five documented outbreaks in NSW since 2014 and 24 individuals affected. These outbreaks were related to Spanish mackerel consumption [15].

These outbreaks of illness show that food safety failures can occur due to the complexity of the supply chain. In most of the cases, poor hygiene and sanitation, lack of temperature control, natural toxins in fish (ciguatera and scombroid), and consumption of raw or undercooked seafood were recorded as contributing factors. Therefore, continuous efforts are needed to monitor the industry practices and improve the existing processes for safer seafood supply in NSW.

## 4. Regulation and Management of Seafood Safety in NSW

**Figure 1:**
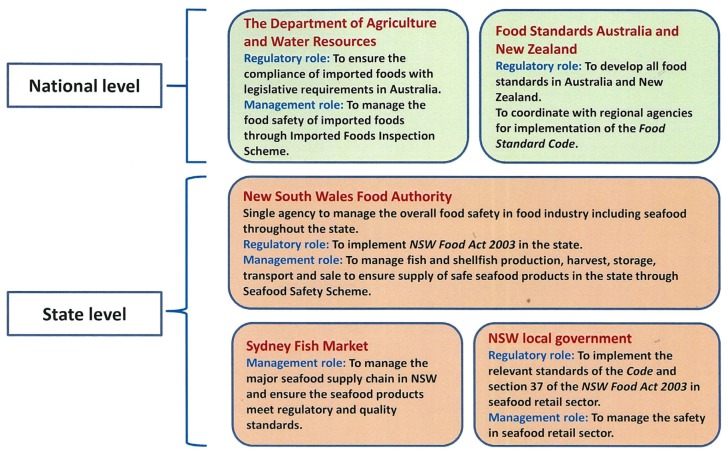
Food safety management and regulatory roles of different agencies in the seafood supply in Australia and New South Wales (NSW).

NSW seafood supply is very well regulated to ensure food safety. The high standard of seafood safety management in NSW is an example of effective coordination between several government agencies and organisations. Several agencies and organisations play key roles in the regulation and management of food safety issues related to seafood industry in NSW (Figure 1). However, the Food Authority plays a vital and major role in the overall government approach to the management of safety challenges faced by food industry including seafood in the state. It also liaises with other stakeholders and agencies to manage seafood safety related issues at the state and national level.

### 4.1. NSW Food Authority

The Food Authority has the main responsibility to manage the overall risks through the Seafood Safety Scheme [16]. The Food Authority’s Seafood Safety Scheme is designed to manage fish and shellfish production, harvest, storage, transport and sale to ensure the supply of a safe product in the state. The NSW Shellfish Program and NSW Marine Biotoxin Management Plan (MBMP) and audit of licensed seafood businesses are examples of specific initiatives to manage food safety related to a particular sector or challenge faced by the seafood industry.

### 4.2. Sydney Fish Market

A large volume of seafood supply in NSW is traded through the Sydney Fish Market (SFM). It is the largest market of its kind in the Southern Hemisphere and the third largest seafood market in terms of variety in the world. On average, 50 tonnes of fresh seafood is traded through SFM every day [17]. SFM is considered as Australia’s seafood centre of excellence and strives for the highest levels of quality and customer satisfaction. SFM has maintained a quality assurance program and Hazard Analysis Critical Control Points (HACCP) system since 1998 to ensure that seafood sold is safe to eat, accurately labelled and satisfies the customer.

### 4.3. The Department of Agriculture and Water Resources

The department has the responsibility to manage the compliance of imported food with the Australian food standards and the requirements of public health and safety. All imported foods are subject to compliance control through legislative tiers; the *Imported Food Control Act 1992* by the Australian Government Attorney-General’s Department, Commonwealth of Australia Law 2009, the Imported Food Control Regulations 1993, and the Imported Food Control Order 2001. The Department of Agriculture and Water Resources (DAWR) uses a number of approaches for managing the safety of imported foods. These approaches include Imported Foods Inspection Scheme (IFIS), foreign government certificates, quality assurance arrangements, and compliance arrangements.

The Imported Food Control Order (2001) specifies risk food that must be inspected, or inspected and analysed under the IFIS. The Order is a list of high risk food categories and some examples from the Order lists are given below:

(1) Crustaceans, including prawns, that are cooked (whether or not chilled or frozen), but are not canned,
(2) The specific kinds of fish (i.e., tuna, including canned tuna (whether dried or not), tuna products, mackerel and ready-to-eat finfish),
(3) Marinara mix (whether or not chilled or frozen), and
(4) Molluscs, bivalve (whether cooked or uncooked).

The food safety assurance of imported food is a joint responsibility of many Australian Government agencies. The DAWR monitors imported seafood as part of a broader food regulatory system. Imported food must meet the Australian food standards as is the case with food produced domestically. Food Standards Australia New Zealand (FSANZ) is responsible for determining and periodically reviewing the list of potential hazards for risk foods through the Imported Food Notices. High risk foods are routinely inspected and analysed for the identification of hazards by DAWR. Post border food safety of imported food is managed by each State and Territory through their relevant state legislation.

### 4.4. Food Standards Australia and New Zealand

FSANZ has an important role in developing all domestic food standards based on scientific/technical criteria, consistent with Ministerial Council policy and advising the DAWR on the risk categorization of foods for the purpose of inspection under the IFIS. The *Australia New Zealand Food Standards Code* (The *Code*) has several standards and requirements that apply to seafood, for seafood produced domestically or imported. The relevant sections of the *Code* pertaining to seafood are:

(1) Standard 4.2.1 Primary Production and Processing Standard for Seafood (PPPS Seafood),
(2) Standard 1.3.1 Food Additives (specific to sulphur dioxide levels),
(3) Standard 1.4.1 Contaminants and Natural Toxicants (specific to histamine levels),
(4) Standard 1.6.1 Microbiological limits for food, and
(5) Standard 2.2.3 Fish and Fish Products

The *Code* also has several other requirements that apply to seafood, for example:

(1) Limits of residues that can be present in seafood from the use of agricultural and veterinary chemicals,
(2) Maximum levels for certain potential chemical contaminants as per the contaminant standard,
(3) Microbiological limits for known human pathogens and other microorganisms that are indicators for human pathogens,
(4) Permission to use certain additives in seafood as per the food additives standard (maximum levels are prescribed for some permissions),
(5) Labelling requirements that apply to all foods, and
(6) Food Safety Standards which apply to seafood businesses, including seafood importers and seafood handlers.

FSANZ has the responsibility to assess food safety risks to consumers and then prioritise seafood commodities for classification as ‘risk foods’.

### 4.5. Seafood Safety Management at Retail Level

The Food Authority’s Seafood Safety Scheme does not cover food safety management at the retail level. The safety of foods in retail is covered by the *Code*. Standard 3.2 of the *Code* specifies the food safety requirements for the handling and sale of the food products through three standards:

(1) Standard 3.2.1 Food safety programs
(2) Standard 3.2.2 Food safety practices and general requirements
(3) Standard 3.2.3 Food premises and equipment

The NSW seafood retail sector is primarily managed by local government environmental health officers to ensure that the businesses comply with the hygiene and good handling practices under Section 37 of the *NSW Food Act 2003*. The Food Authority has developed a guideline [18] to assist seafood retailers in meeting the food safety and labelling requirements outlined in the *Code*, which is the law in Australia.

## 5. Conclusions

NSW has a dynamic seafood industry that ensures the supply of a variety of seafood products. Food safety of the seafood supply is jointly regulated and managed by several agencies where the Food Authority plays a major role. The Food Authority adopts a holistic approach to manage overall risks through implementation of the Seafood Safety Scheme, targeted programs to control specific hazards, and partnering with the local government sector that inspects restaurants and cafes. In general, seafood products pose low risk to the consumers due to the small number of average outbreaks (about 4) that occur per year in NSW.

## Figures and Tables

**Table 1:** Profile of New South Wales (NSW) seafood industry 2012–2013 [5].

	Total	Wild Catch	Aquaculture
Production (tonnes)	15,783	11,597 (73.48%)	4186 (26.52%)
Value (Aus$)	123.7 million	76.2 million (61.58%)	47.5 million (38.42%)
Key species	Finfish Prawns Crabs Lobsters Oysters	Finfish: snapper, yellowfin bream, flathead, sea mullet, ocean jacket, yellowtail kingfish, blue-eye trevalla. Prawns: eastern king prawns, school prawn, black tiger prawns. Crabs: spanner crab, blue swimmer crab, mud crab. Lobster: Eastern rock lobster.	Finfish: silver perch, snapper, yellowtail kingfish, mulloway, rainbow trout, barramundi. Prawns: tiger prawns. Oysters: Sydney rock oysters, Pacific oysters, native oysters.

**Table 2:** Summary of NSW foodborne illness outbreaks attributed to seafood (2005–2015).

	Hazard	Outbreaks	Cases	Hospitalisations
Seafood total	Ciguatoxin	4	21	14
	Scombroid	11	35	19
	*Salmonella* non-typhi ^1^	9	41	9
	Norovirus	3	22	0
	Others-bacterial	2	35	2
	Unknown	14	154	1
	Total	43	308	45
Finfish	Ciguatoxin	4	21	14
	Scombroid	11	35	19
	*Salmonella* non-typhi ^1^	8	37	7
	Others-bacterial	2	35	2
	Unknown	3	10	1
	Sub-total	28	138	43
Shellfish	Norovirus	3	22	0
	Unknown	7	43	0
	Sub-total	10	65	0
Crustacean total	*Salmonella* non-typhi ^1^	1	4	2
	Unknown	4	101	0
	Sub-total	5	105	2

^1^ Cross-contamination of seafood from egg when used as an ingredient. Data source: NSW OzFoodNet (a health network to enhance the surveillance of foodborne diseases in Australia).
